# msPIPE: a pipeline for the analysis and visualization of whole-genome bisulfite sequencing data

**DOI:** 10.1186/s12859-022-04925-2

**Published:** 2022-09-19

**Authors:** Heesun Kim, Mikang Sim, Nayoung Park, Kisang Kwon, Junyoung Kim, Jaebum Kim

**Affiliations:** Department of Biomedical Science and Engineering, Konkuk University, Seoul, 05029 Republic of Korea

**Keywords:** DNA methylation, Pipeline, Whole-genome bisulfite sequencing, Next generation sequencing

## Abstract

**Background:**

DNA methylation is an important epigenetic modification that is known to regulate gene expression. Whole-genome bisulfite sequencing (WGBS) is a powerful method for studying cytosine methylation in a whole genome. However, it is difficult to obtain methylation profiles using the WGBS raw reads and is necessary to be proficient in all types of bioinformatic tools for the study of DNA methylation. In addition, recent end-to-end pipelines for DNA methylation analyses are not sufficient for addressing those difficulties.

**Results:**

Here we present msPIPE, a pipeline for DNA methylation analyses with WGBS data seamlessly connecting all the required tasks ranging from data pre-processing to multiple downstream DNA methylation analyses. The msPIPE can generate various methylation profiles to analyze methylation patterns in the given sample, including statistical summaries and methylation levels. Also, the methylation levels in the functional regions of a genome are computed with proper annotation. The results of methylation profiles, hypomethylation, and differential methylation analysis are plotted in publication-quality figures. The msPIPE can be easily and conveniently used with a Docker image, which includes all dependent packages and software related to DNA methylation analyses.

**Conclusion:**

msPIPE is a new end-to-end pipeline designed for methylation calling, profiling, and various types of downstream DNA methylation analyses, leading to the creation of publication-quality figures. msPIPE allows researchers to process and analyze the WGBS data in an easy and convenient way. It is available at https://github.com/jkimlab/msPIPE and https://hub.docker.com/r/jkimlab/mspipe.

**Supplementary Information:**

The online version contains supplementary material available at 10.1186/s12859-022-04925-2.

## Background

DNA methylation entails attachment of methyl groups to a base of DNA, especially cytosine in the dinucleotide CpG sites. DNA methylation regulates gene expression and contributes directly to disease conditions. For example, DNA methylation directly affects carcinogenesis [1, 2] and genomic imprinting or X chromosome inactivation [3]. During mammalian development, the imprinting of one of the paternal or maternal chromosomes induces differential expression of imprinted genes, and the loss of imprinting can cause various genetic diseases in humans [4], Beckwith–Wiedemann syndrome [5], and Prader–Willi syndrome [6]. Furthermore, DNA hypermethylation, which refers to an increased level of DNA methylation, involving CpG islands of certain genes may result in the silencing of tumor suppressor genes [7, 8]. Recent studies have reported that DNA hypomethylation, which involves the loss of methyl groups, occurs in carcinogenesis and tumor progression [9]. In addition, hypomethylation in repetitive regions of a genome can contribute to genomic instability, such as transposon reactivation or homologous recombination [10]. While 70–90% of CpG dinucleotides are methylated in normal cells of human tissues [11], most of the CpG islands in promoters are unmethylated [12]. Methylation of the CpG islands results in gene silencing and regulates gene expression during development and differentiation [13].

Many methods have been developed for DNA methylation sequencing, including those based on restriction enzymes [14], affinity enrichment [15, 16], and bisulfite conversion [17, 18]. The bisulfite conversion-based methods are commonly used to identify and quantify DNA methylation using several next-generation sequencing technologies, including whole-genome bisulfite sequencing (WGBS) [19, 20] and reduced representation bisulfite sequencing (RRBS) [21]. Specifically, WGBS can be used to identify DNA methylation of cytosines in entire genome, while RRBS can be used only for small segments in a genome.

DNA methylation analysis using WGBS has been performed via multiple steps, including read quality control, read mapping to reference genome sequences, and methylation calling. Computational tools such as TrimGalore! [22] and FastQC [23] are used to remove adaptor sequences and control read quality. WGBS reads are mapped to reference genome sequences using specific mapping tools, such as Bismark [24] and BS Seeker [25], which can generate and use converted reference genome sequences for accurate processing of converted unmethylated cytosine bases in the reads. Methylation calling tools such as Bismark [24] and Bicycle [26] compute the methylation level, which represents the degree of methylation for all mapped cytosines. Additionally, some R packages, such as methylKit [27] and MethylSeekR [28] can be used to identify differentially methylated cytosines (DMCs) and hypomethylated regions from the methylation calls.

Despite the development of the computational tools dealing with sequencing data, studying DNA methylation is still a research challenge because it requires the knowledge of bioinformatics. The investigators also need to select and organize appropriate reference genome sequences and their annotation information because most of the DNA methylation analyses are performed using the reference genome. To alleviate these difficulties, several pipelines have been developed to analyze methylation sequencing data [27, 29–32]. However, various types of DNA methylation analyses are not always available in those pipelines, and the available reference genomes and tools are restricted. For example, as shown in Table 1, most of them [26, 33–37] do not completely support important downstream analyses, such as the analyses of differentially methylated and hypomethylated regions. Moreover, they are mostly focusing on analyzing human and it is difficult to be applied for other species because required genome sequences and annotation information of a reference need to be prepared and set manually.

**Table 1:** Comparison of methylation analysis pipelines

Pipeline	Installation	Quality control	Alignment	Methylation calling	DMC/DMR analysis	HMR analysis	Gene function analysis	Reference setting
msPIPE	Docker Manual	Cutadapt Trim Galore! MultiQC	Bismark BS-Seeker2	Bismark BS-Seeker2	methylKit BSmooth	MethylSeekR	g:Profiler	Automatic^a^
BAT [33]	Docker Manual	BAT	segemehl	haarz	metilene	NA	NA	Manual
bicycle [26]	Manual Docker Live CD	bicycle	bicycle	bicycle	bicycle	NA	NA	Manual
ENCODE pipeline [34]	DNAnexus	Trim Galore! SAMtools Bismark	Bismark	Bismark	NA	NA	NA	Manual
Msuite [35]	Manual	Msuite	Msuite	Msuite	NA	NA	NA	Manual
Nextflow methylseq (Bismark) [36]	Nextflow	Trim Galore! MultiQC	Bismark	Bismark	NA	NA	NA	Automatic^b^
Nextflow methylseq (bwa-meth) [36]	Nextflow	Trim Galore! MultiQC	bwa-meth	MethylDackel	NA	NA	NA	Automatic^b^
PiGx BS-seq [37]	GNU guix	Trim Galore! MultiQC	Bismark	methylKit	methylKit	NA	NA	Manual
snakePipes [46]	Bioconda	Cutadapt Trim Galore! Fastp MultiQC	bwa-meth	MethylDackel	dmrseq DSS metilene	NA	NA	Partially automatic^c^
wg-blimp [47]	Bioconda Docker	MultiQC	bwa-meth	MethylDackel	bsseq camel metilene	MethylSeekR	NA	Manual

*NA* not available

^a^All required files of a reference can be automatically prepared and set if the data exists in the UCSC Genome Browser database [40], and manual setting is also supported

^b^All required files of a reference can be automatically prepared and set if the data exists in the iGenomes database [48], and manual setting is also supported

^c^All required files of a reference can be automatically prepared and set if the reference is one of five species (human, mouse, zebrafish, fruit fly, and fission yeast), and manual setting is also supported

In this study, we present a new end-to-end pipeline of DNA methylation analysis for WGBS data. This pipeline, named msPIPE, consists of multiple steps: (i) pre-processing, (ii) alignment & methylation calling, and (iii) methylation analysis & visualization. Using msPIPE, users can obtain the results of various analyses in text format and publication-quality figures. Additionally, the use of msPIPE is facilitated by Docker, which obviates the need for requisite packages or software. The msPIPE supports all reference genome assemblies available in the R package BSgenome (v1.62.0) [38]. Applications involving human and mouse WGBS datasets successfully utilized the msPIPE to generate methylation profiles of human sperm and mouse rod samples, profiles of genomic context, and differentially methylated regions, with additional findings by the functional enrichment analysis. The msPIPE will facilitate our understanding of DNA methylation in the targeted species and interpretation of DNA methylation-based studies.

## Implementation

**Fig. 1:**
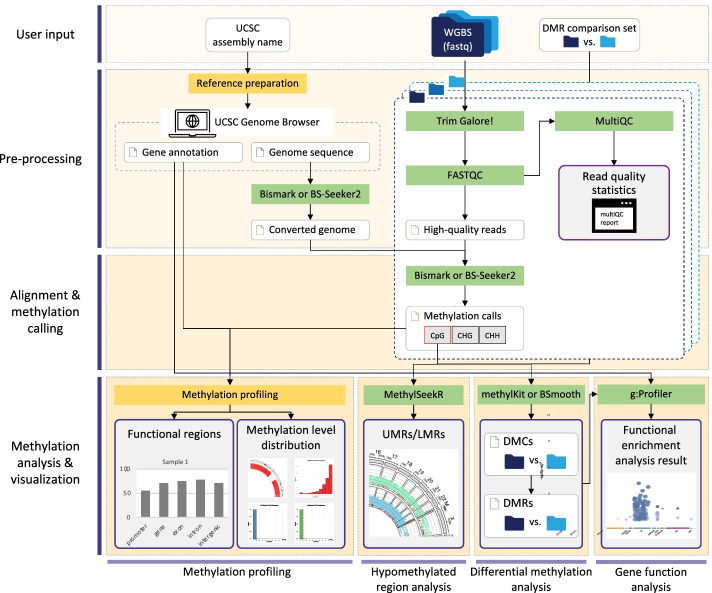
Overview of the msPIPE workflow. Using WGBS read files and UCSC assembly name of a reference as input, the msPIPE automates the entire DNA methylation analysis starting from input data pre-processing to methylation analysis. The reference genome sequences and annotation files of input species are collected from the UCSC genome browser. The trimmed reads are mapped to the bisulfite-converted genome sequences, and methylation calls are made. Based on these methylation calls, methylation profiling, hypomethylated regions analysis, differential methylation analysis, and the function analysis for methylation-related genes are performed

The msPIPE pipeline consists of pre-processing, alignment & methylation calling, and methylation analysis & visualization steps (Fig. 1). It generates a DNA methylation profile for each sample, which is a unit of analysis defined by user. The msPIPE can be used to treat one or more replicates for each sample. In brief, the required reference files are prepared using the given UCSC assembly name of a reference, and the input bisulfite sequencing reads in each sample are trimmed first. The pre-processed reads are then mapped to the bisulfite-converted reference genome sequences, and methylation calls are obtained for each cytosine context. Based on the identified methylation calls for all replicates derived from each sample, the sample-level merged methylation coverages are computed to generate methylation profiles for the given samples for downstream methylation analyses. The analyses of hypomethylated and differentially methylated regions are performed using the methylation calls and profiles. For the genes related to differentially methylated cytosines, functional analysis is additionally performed. Finally, the results of DNA methylation analyses included are visualized, and publication-quality figures are created.

The msPIPE is implemented using Python, Perl, and R. The pipeline requires other software programs; however, the user can easily install and use it via Docker image [39]. This Docker image, which contains all dependent packages and software, can be obtained by loading image files from the Docker Hub or building directly by using the Docker files provided in the Github site.

### Pre-processing step

In this step, input data for methylation analysis is prepared and required pre-processing is done. This step consists of preparing the reference genome sequences, annotation files, and high-quality WGBS reads for analyses. For the given UCSC assembly name of a reference, a corresponding genome sequence file and a gene annotation file are automatically downloaded from the UCSC Genome Browser database [40]. Note that users can also use their own genome sequences or annotation file as desired.

The msPIPE can receive multiple fastq files of single-end or paired-end reads for all sample replicates. All input fastq files are processed separately given that WGBS reads are trimmed by TrimGalore! (v0.6.0) [22] using ‘--fastqc --phred33 --gzip --length 20’ options and the quality of sequencing reads can be determined using FastQC (v0.11.9) [23]. The quality reports of trimmed reads are illustrated with a merged report by the MultiQC program (v1.10) [41].

The downloaded reference genome sequences are changed to bisulfite-converted reference genome sequences by converting C to T and G to A using Bismark (v0.20.0) (the bismark_genome_preparation module) [24] or BS-Seeker2 (the bs_seeker2-build.py module) [42]. To reduce running time, the bisulfite-converted reference genome sequences can be reused in the next run with the same UCSC assembly name of a reference as the input.

### Alignment and methylation calling

In the alignment step, pre-processed WGBS read sequences are aligned to the bisulfite-converted reference genome sequences. For this alignment, two programs, Bismark [24] with ‘--score_min L,0,-0.6 -N 0 -L 20’ options and BS-Seeker2 [42] with ‘-m 0’ option, are supported.

In the methylation calling step, methylated genomic regions are identified by using the alignments of the WGBS read sequences. When Bismark is used, methylation calls are generated for CpG, CHG, and CHH context using the bismark_methylation_extractor module with ‘--no_overlap --comprehensive --gzip --CX --cytosine_report’ options based on the WGBS read mapping files generated in the previous step. Alternatively, if BS-Seeker2 is used, methylation calls are generated for all CX contexts using the bs_seeker2-call_methylation.py program with ‘--sorted --rm-overlap’ option. The output files of BS-Seeker2 were then converted to the files with the same format as the ones of Bismark using in-house Python script.

### Methylation analysis and visualization

In this step, called methylated regions are used to perform downstream analyses and visualize their results. The methyl-C calls obtained in the previous steps with CX (CpG, CHG, and CHH) context are used as input for various methylation analyses and the creation of publication-quality figures. This step consisting of three sub-steps, methylation profiling, hypomethylated region analysis, and differential methylation analysis, as described below.

### Methylation profiling

In this sub-step, two types of methylation profiles including methylation patterns for CX context and functional annotated regions are analyzed and plotted for each target sample. For this, sample-level merged methylation calls are generated by combining all methyl-C calls from all sample replicates at the same position on the reference genome. First, using the sample-level merged methylation calls, methylation profiles are obtained by calculating methylation levels for each CX context. The methylation level for each base position comprising methylation calls is defined by the ratio of the counts of methylated Cs to the total counts of both methylated and unmethylated Cs. These methylation profiles are plotted as a bar plot with the average methylation level, and as a histogram with the methylation level distribution of each CX context for each sample. Whole genome-scale methylation profile is plotted into the Circos plot with three different tracks using the R package circlize (v0.4.13) [43]. The average methylation level in a bin (bin size: 100 Kbp) is calculated for all chromosomes and displayed as the outermost track in the genome-wide profiling Circos plot [43]. Second, five genomic contexts, including promoter, gene, exon, intron, and intergenic regions, are defined for the entire genome from a downloaded UCSC gene annotation file to determine the methylation patterns of the functional annotated regions. Specifically, a promoter is defined as a 1 Kbp upstream region of a gene. Exons are defined as merged regions annotated as an exon from all transcripts, and introns are defined by excluding the exon portions from the genes. For every gene, average methylation levels in a sliding window (window size: 500 bp and step size: 100 bp) from 1500 bp upstream to 1500 bp downstream of a transcription start site (TSS) are calculated. Finally, the regions excluding the transcript areas from the entire genome are defined as the intergenic regions. The degree of methylation distribution for each CX context and functional annotated region are visualized by the R package ggplot2 (v3.3.5) [26].

### Hypomethylated region analysis

In this sub-step, hypomethylated regions (HMRs), which are contiguous genomic regions with lower methylation level than neighboring regions, are identified by using the R package MethylSeekR (v1.34.0) [28] by automatically selecting the BSgenome data package using the UCSC assembly name of a reference given as the input of msPIPE. Two different types of HMRs include unmethylated regions (UMRs), which are enriched in CpG with almost zero methylation levels, and low methylated regions (LMRs), which are CpG-poor regions with low methylation (around 30%). The coordinates of UMRs and LMRs are reported as text files and their locations are plotted into the middle and innermost tracks, respectively, in the Circos plot indicated in the methylation profiling sub-step. In addition, the coordinates and counts of UMRs, which overlap with promoters are reported as the bed format files, which facilitates identification of methylation patterns in the target genes.

### Differential methylation and gene function analysis

When pairs of samples for pairwise comparison of methylation are set, the analysis of differentially methylated regions (DMRs) is performed for each comparison set in this sub-step. DMRs are defined as the genomic regions with different methylation level between two samples which can be called as case and control respectively. DMRs can be further divided into hypomethylated (with relatively lower level of methylation) or hypermethylated (with relatively higher level of methylation) regions. Specifically, when the methylation level of a genomic region in the case is lower than the level in the control, the genomic region in the case is defined as the hypomethylated DMR. In the opposite case, the genomic region in the case is defined as the hypermethylated DMR. The analysis of DMRs can be performed using either methylKit [27] or BSmooth [44]. The definition of DMRs is different in the two programs. In the case of methylKit, a genomic region harboring two or more differentially methylated (either hypomethylated or hypermethylated) Cs (DMCs) with a maximum 500 bp distance between two adjacent DMCs is considered as a DMR. In BSmooth, a DMR is defined as a genomic region which has three or more DMCs with a minimum 10% methylation difference and covers at least 70 CpGs (minimum length 1 Kbp). Methylation differences are reported for each methyl-C position with a q-value. The msPIPE can filter out the methyl-C positions with the q-value less than the given cutoff. Various features of generated DMCs, including the number and genomic locations of hypomethylated and hypermethylated Cs in promoters, distance between DMCs, and distance between DMC and nearest TSS are summarized. Additionally, a list of DMC (or DMR)-related genes is created by collecting genes whose promoter region contains the DMC (or DMR) and used for input of g:Profiler [45] which is a functional enrichment analysis tool.

## Results and discussion

### Comparison of msPIPE with similar pipelines for methylation analysis

msPIPE and similar pipelines for methylation analysis [26, 33–37, 46, 47] were compared in terms of installation, supported sub-steps, downstream analyses, and the difficulty of setting a reference (Table 1). Most of them can be easily installed by using a cross-platform and dependency-free package manager, such as Docker and Bioconda. Three basic sub-steps for methylation analysis, which are quality control, alignment and methylation calling, are supported by all compared pipelines. However, in the alignment and methylation calling sub-steps, only msPIPE supports two optional tools, Bismark and BS-Seeker2, which increases the flexibility of users for trying different tools and compare their results for drawing better conclusion. msPIPE and wg-blimp are the only pipelines that can be used for the downstream analyses for both of differentially methylated and hypomethylated regions, and only msPIPE supports the function analysis of methylation-related genes. Nextflow methylseq and msPIPE are the only pipelines that can automatically prepare and set the required genome sequences and annotation information of a reference based on the iGenomes [48] and UCSC Genome Browser database [40] respectively. In summary, msPIPE is better than the compared nine pipelines in terms of supported optional tools, additional downstream analyses, and the convenience of preparing reference genome data.

### Application of msPIPE to human and mouse WGBS datasets

The msPIPE was applied to publicly-available human (accession number: PRJEB28044) [49] and mouse (accession number: PRJNA556668) WGBS datasets [50] (Additional file 2: Table S1). The human dataset was generated from the pooled libraries of DNA obtained from the blood and sperm of six young men (18–24 years) and six old men (61–71 years), respectively. The human genome assembly version hg38 was used as the reference genome [51]. The mouse dataset was generated from rod photoreceptors belonging to three young (three-month-old) and three old (24-month-old) male mice. For reference genome, the mouse genome assembly version mm10 was used [52]. The msPIPE was executed for the human and mouse datasets with default options except for the ‘-c 5 -q 0.5’ option. In this application, Bismark was used for alignment & methylation calling and methylKit was used for DMC analysis. A list of genes with promoters carrying DMC was extracted, followed by functional enrichment analysis using g:Profiler [45].

### Methylation profiles of human and mouse WGBS data generated by msPIPE

To assess the applicability of msPIPE, methylation analyses were performed using the published human and mouse WGBS datasets (see Implementation). Pre-processing, mapping to bisulfite-converted genome sequences, and methyl-C calling were performed sequentially.

**Fig. 2:**
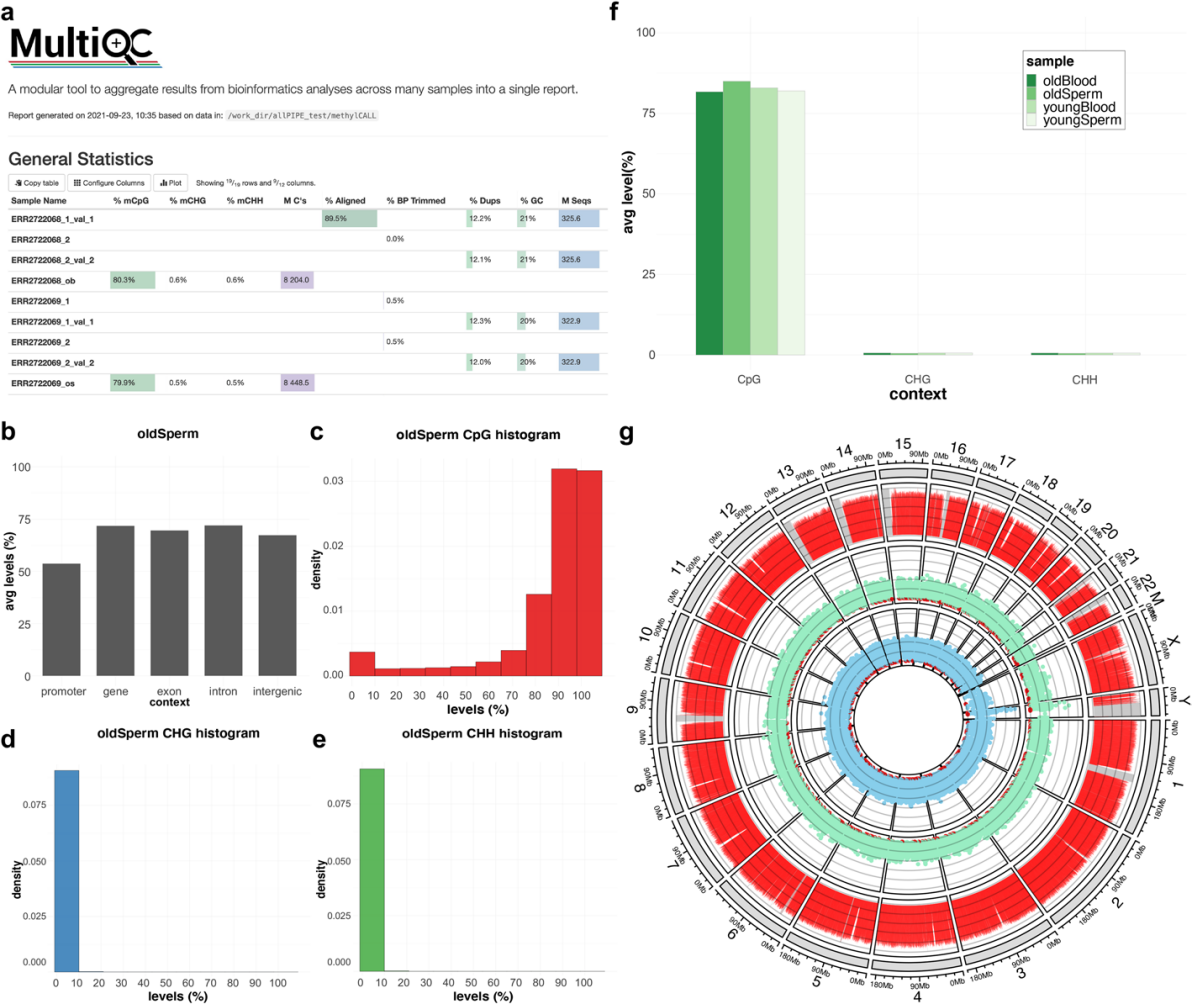
Sample results of msPIPE using the human WGBS dataset. **a** The read quality and statistics of all processed input samples were reported to the MultiQC html file. **b** The average CpG methylation levels in each genomic context, including promoter, gene, exon, intron, and intergenic regions of the old sperm sample are represented by a bar plot. The methylation levels (%) of **c** CpG, **d** CHG, **e** CHH context in the old sperm sample are shown. The bin size of the histogram is 10%. **f** The average levels of CpG, CHG, and CHH methylation for each given sample. **g** Genome-wide CpG methylation levels as well as UMR and LMR distribution in the old sperm sample are presented as the Circos plot. The red bar plot on the outermost track represents the average methylation level for 100 Kbp bin. In the absence of data, it was represented by a gray shadow. The dot plots on the inner two tracks represent UMR region shown in light green and the LMR region in light blue. The height of the graph indicates the methylation level of each region. A zero average methylation of the UMR (or LMR) is indicated by a red dot

Trimming for adaptor and low-quality sequences was performed for all input WGBS reads. The quality of each trimmed read was computed using FastQC program. Additionally, all reports were summarized as an html file using MultiQC program. For example, the statistical data of read quality for all human and mouse WGBS reads after pre-processing were summarized together (Fig. 2a; Additional file 1: Fig. S1a).

After pre-processing, the various methylation profiles for human and mouse samples were generated. First, the methylation level of each C called using the Bismark package was calculated and summarized with genomic and CX context. For the genomic context, the average methylation levels of each functional region were obtained. For the human old sperm dataset, the average methylation level of the promoter, mostly known to be located in CpG islands [13], was lower than in other genomic contexts (Fig. 2b). Additionally, the distribution of methylation levels for CX contexts was calculated and plotted. CHG and CHH contexts were hardly methylated in the human old sperm dataset (Fig. 2c–e) and were clearly detected in the mouse dataset (Additional file 1: Fig. S1b–f). The average methylation level of CX contexts was also calculated and summarized together with all samples (Fig. 2f). Hypomethylated regions for each sample were predicted, and the UMRs and LMRs were distributed in all chromosomes (Fig. 2g; Additional file 1: Fig. Sg; UMRs for blue, and LMRs for green color).

We performed the differential methylation analysis between control and case as young versus old for all three pairs of samples: human blood, human sperm, and mouse rod. In the human dataset, the total number of DMCs with a q-value of 0.5 or less was 244 in blood and 34,514 in sperm samples (Additional file 2: Tables S2 and S3). Based on mouse data, 274 DMCs were predicted (Additional file 2: Table S4).

### Differentially methylated genes associated with strong sperm mobility in humans identified in msPIPE outputs

In the previous study for the human WGBS dataset [49], gene ontology analysis of DMR related gene set was conducted using the list of genes in the 1 Mbp region upstream and downstream of DMR. This analysis revealed significant enrichment of the 121 hypomethylated DMR neighboring genes in the homeobox, DNA bonds, nuclei, and transcription.

The results of human DMC analyses derived from msPIPE revealed additional findings. Among the human sperm samples, 393 differentially methylated genes with one or more DMCs in the promoter were identified (Additional file 2: Table S5). The functional enrichment analysis of 393 differentially methylated genes [45] revealed enrichment of GO terms for metal ion binding (GO:0046872) and cation binding (GO:0043169), and transcription factors for many other genes including SRY (Table 2; Additional file 2: Table S6). Notably, the influx of cations through cation channels (CatSper) is known to play an important role in fertility and motility of sperms [53, 54]. Highly specific and important candidate genes were identified with promoter methylation patterns based on DMC analysis of msPIPE.

**Table 2:** Functional enrichment analysis results for 393 differentially methylated genes in human sperm samples

Source	Term name	Term id	Adjusted p value*
GO:MF	Metal ion binding	GO:0046872	8.215E−03
GO:MF	Cation binding	GO:0043169	1.289E−02
TF	Factor: SRY; motif: TCAATAMCATTGA	TF:M04557	9.270E−10
TF	Factor: SRY; motif: AACAATNNNCATTGTT	TF:M04556	7.598E−07
TF	Factor: SRY; motif: AACAATNNNCATTGTT; match class: 1	TF:M04556_1	5.787E−05
TF	Factor: SRY; motif: TCAATAMCATTGA; match class: 1	TF:M04557_1	6.900E−05
TF	Factor: SRY; motif: AACAATANCATTGTT	TF:M04555	2.568E−04
TF	Factor: SRY; motif: TTGTTT; match class: 1	TF:M03854_1	8.544E−04
TF	Factor: SRY; motif: AACAATNR; match class: 1	TF:M08976_1	1.131E−03
TF	Factor: SRY; motif: AACAATANCATTGTT; match class: 1	TF:M04555_1	2.291E−03
TF	Factor: SRY; motif: AACAATNR	TF:M08976	1.300E−02
TF	Factor: SRY; motif: TTGTTT	TF:M03854	1.750E−02

*Adjusted p value was calculated by the g:SCS method in g:Profiler

## Conclusion

We present an end-to-end WGBS analysis pipeline, msPIPE, used to perform bioinformatic analyses ranging from input read pre-processing to downstream analysis. When the input WGBS read sequencing files and the UCSC assembly name of a reference are given, the user can conveniently obtain methylation profiles, publication-quality figures, differentially methylated regions, and related genes for a given comparison pair. In the comparison with existing nine pipelines, msPIPE was found to perform better in terms of supported types of analyses, supported optional tools, and a convenient way for the preparation of reference genome data. The msPIPE is implemented using the Docker image, which obviates the need to install all dependent packages and software. Especially, specific R packages dependent on different UCSC assembly versions of references for all kind of species provided by the R package BSgenome are automatically imported along with sets for running msPIPE. Therefore, msPIPE can be used as a convenient and effective tool for methylation analysis of WGBS data.

## Supplementary Information

**Additional file 1.** Supplementary figures.

**Additional file 2.** Supplementary tables.

## Data Availability

WGBS datasets used as input for msPIPE are available in the NCBI SRA) with accession numbers ERR2722068, ERR2722069, ERR2722070 and ERR2722071 for human, and SRR9833662, SRR9833663, SRR9833664, SRR9833670, SRR9833671, and SRR9833672 for mouse. **Availability and requirements** Project name: msPIPE; Project home page: https://github.com/jkimlab/msPIPE; Operating system(s): Linux; Programming language: Python, Perl, R; License: MIT; Any restrictions to use by non-academics: license needed.

## Abbreviations

WGBS: Whole-genome bisulfite sequencing
RRBS: Reduced representation bisulfite sequencing
GO: Gene ontology
DMC: Differentially methylated C
TSS: Transcription start site
HMR: Hypomethylated region
UMR: Unmethylated region
LMR: Low-methylated region
DMR: Differentially methylated region
