# Supplementary material for: msPIPE: a pipeline for the analysis and visualization of whole-genome bisulfite sequencing data
Source: BMC Bioinformatics. 2022 Sep 19;23:383. doi: 10.1186/s12859-022-04925-2 (PMC9487059; doi:10.1186/s12859-022-04925-2)
Supplement: Supplementary file 1 — Additional file 1. Supplementary figures. [file 12859_2022_4925_MOESM1_ESM.pdf]

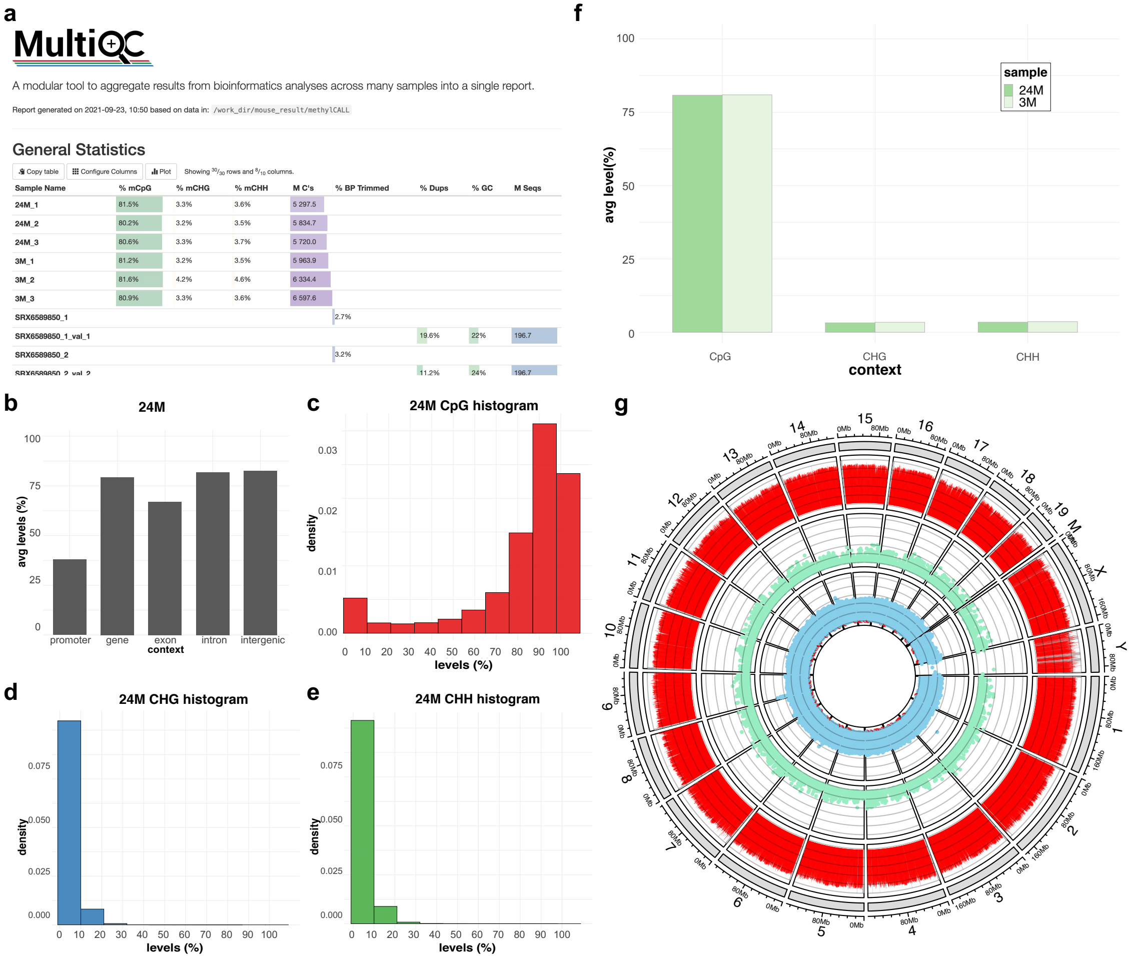
a
MultiQC
A modular tool to aggregate results from bioinformatics analyses across many samples into a single report.
Report generated on 2021-09-23, 10:50 based on data in: /work_dir/mouse_result/methylCALL
General Statistics
Copy table
Configure Columns
Plot
Showing 30/30 rows and 8/10 columns.
Sample Name | % mCpG | % mCHG | % mCHH | M C's | % BP Trimmed | % Dups | % GC | M Seqs
24M_1 | 81.5% | 3.3% | 3.6% | 5 297.5
24M_2 | 80.2% | 3.2% | 3.5% | 5 834.7
24M_3 | 80.6% | 3.3% | 3.7% | 5 720.0
3M_1 | 81.2% | 3.2% | 3.5% | 5 963.9
3M_2 | 81.6% | 4.2% | 4.6% | 6 334.4
3M_3 | 80.9% | 3.3% | 3.6% | 6 597.6
SRX6589850_1 | 2.7%
SRX6589850_1_val_1 | 19.6% | 22% | 196.7
SRX6589850_2 | 3.2%
SRX6589850_2_val_2 | 11.2% | 24% | 196.7
f
sample
24M
3M
avg level(%)
100
75
50
25
0
CpG
CHG
CHH
context
b
24M
avg levels (%)
100
75
50
25
0
promoter
gene
exon
intron
intergenic
context
c
24M CpG histogram
density
0.03
0.02
0.01
0.00
0 10 20 30 40 50 60 70 80 90 100
levels (%)
g
1 2 3 4 5 6 7 8 9 10 11 12 13 14 15 16 17 18 19 M X Y
0Mb
80Mb
160Mb
d
24M CHG histogram
density
0.075
0.050
0.025
0.000
0 10 20 30 40 50 60 70 80 90 100
levels (%)
e
24M CHH histogram
density
0.075
0.050
0.025
0.000
0 10 20 30 40 50 60 70 80 90 100
levels (%)
